# Research and application of digital measure management and control technology for characteristic low-efficiency gas wells in Gas Field A

**DOI:** 10.1371/journal.pone.0323644

**Published:** 2025-05-13

**Authors:** Hao-yang Li, Wen-hai Ma, Jun-liang Li, Cheng-gang Jiang, Pin-gang Ma, De-song Yao, Ming-xi Feng, Shao-xing Gu

**Affiliations:** 1 Daqing Oilfield Production Technology Institute, Daqing, Heilongjiang, China; 2 Heilongjiang Provincial Key Laboratory of Oil and Gas Reservoir Stimulation, Daqing, Heilongjiang, China; 3 State Key Laboratory of Continental Shale Oil, Daqing, Heilongjiang, China; Universiti Teknologi Petronas: Universiti Teknologi PETRONAS, MALAYSIA

## Abstract

Gas Field A has entered the middle and late stages of development, with the number of low-efficiency wells increasing year by year. In the Songliao old area, extreme cold weather (-40°C) has led to 45% of gas wells experiencing freeze-offs. In the Sichuan-Chongqing exploration area, high formation water volume and salinity (35 × 10^4^ mg/L) have resulted in 44% of wells suffering from liquid loading. The annual demand for thawing and foam drainage measures reaches 3,000 well interventions. The large workload and high costs of maintenance make it difficult to ensure the timing and frequency of interventions, affecting the gas recovery efficiency. By establishing an integrated analysis and remote monitoring platform that combines “condition diagnosis, measure adjustment, and remote monitoring,” the use of “freeze-off prediction + precise chemical injection” has improved the opening rate of freeze-off wells. The application of “optimized foam drainage injection parameters” ensures the stable production of liquid-loaded wells. The implementation of this technology is expected to generate over 20 million yuan in benefits and enhance the analysis, decision-making, and control capabilities of gas production processes under extreme weather and production conditions.

## 1. Introduction

In 2023, the natural gas production of Oil and Gas Field A reached a new milestone of 5.85 billion cubic meters, with production from existing wells accounting for 78.5% of the total. The stable production from these existing wells has become the “ballast stone” for the rapid increase in natural gas output. The production blocks are primarily divided into the Songliao old area and the Sichuan-Chongqing exploration area. The Songliao old area, being the earliest developed gas field, experiences extreme winter cold with temperatures dropping to -40°C. The sudden temperature drop during extreme cold weather leads to hydrate freeze-offs in surface pipelines and wellheads, making it difficult to predict the timing of hydrate formation and implement timely deblocking measures. As a result, 83 wells (accounting for 45% of the low-efficiency wells in Songliao) are affected by freeze-offs, impacting gas production by 155.51 million cubic meters annually. The Sichuan-Chongqing area, a newly developed exploration region in recent years, covers a total area of 16,200 square kilometers with only 209 production wells. Most of these wells have entered the late stage of development, characterized by high water production from the reservoir and salinity levels as high as 35 × 10^4^mg/L. The number of liquid-loaded wells has reached 92 (accounting for 44% of the low-efficiency wells in Sichuan-Chongqing). The vast area and sparse well distribution have led to an annual increase in intervention workload. Relying solely on manual management, the maintenance workload is heavy and costly, making it difficult to ensure the timing and frequency of interventions, thereby affecting gas production by 37.58 million cubic meters annually. The distinct characteristics of low-efficiency wells in these two regions necessitate the integration of current digital management methods. By utilizing algorithmic models to predict the timing and location of freeze-offs in advance and remotely adjusting parameters for precise antifreeze injection, the technological gap in “freeze-off management during extreme cold weather” can be addressed. Additionally, by optimizing foam drainage parameters based on digital methods and tailoring the concentration and frequency of foam drainage agent injection to the high salinity conditions, continuous and efficient remote injection of foam drainage agents can be achieved, filling the technological gap in “management of liquid-loaded wells with high salinity.”

## 2. Research on digital control and management technology for inefficient wells

**Fig 1:**
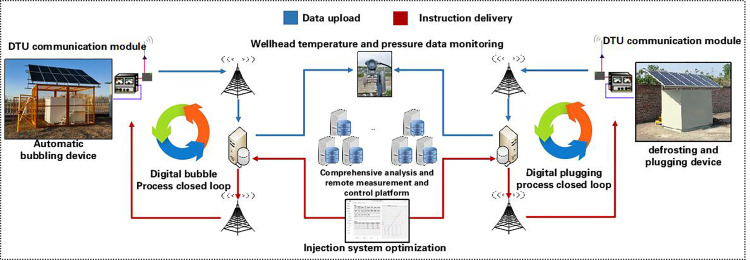
Technology roadmap for digital control of low efficiency wells.

Currently, Gas Field A is in the initial stage of digitalization. To enhance the level of gas production process analysis, decision-making, and management, an integrated analysis and remote monitoring and control platform has been established [1], which combines “condition diagnosis, measure adjustment, and remote monitoring.” The platform utilizes front-end temperature and pressure sensors to capture wellhead production data, employs the oilfield VPDN private network transmission channel for data return, and determines the “cause of issues” through condition diagnosis algorithm models. It dynamically adjusts parameters and optimizes measures using decision-making models to clarify “treatment methods,” thereby enabling remote control of automated chemical injection devices at the wellhead. By analyzing real-time wellhead production data throughout the entire process and optimizing the adjustment of measures, a new model of digital closed-loop control for measures has been established, transforming “manual maintenance” into “digital control.” (Fig 1). This model focuses on the supervision of measures such as freeze blockage and liquid accumulation in wells [2,3].

### 2.1. Research on big data-based operating condition diagnosis and early warning algorithm

Following the principles of data “collection, storage, management, and application,” clarify the flow and application scope of various data types. Process 340,000 real-time data points daily by establishing data collaboration techniques to enable data cleaning, filtering, and extraction of valid data. Utilize the GBDT (Gradient Boosting Decision Tree) algorithm to label and classify gas well operating conditions.

**Fig 2:**
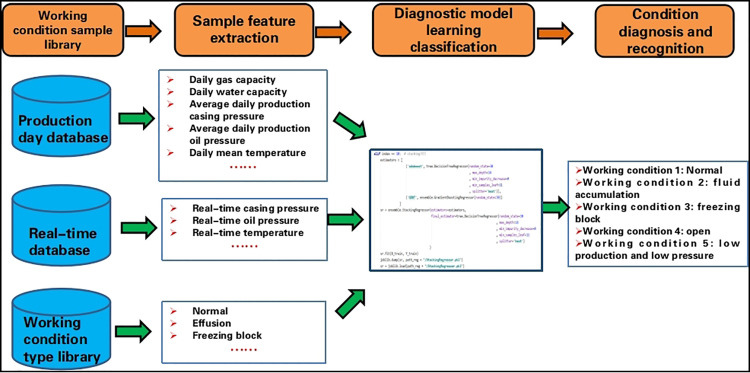
The GBDT diagnosis algorithm model is constructed.

Using the GBDT algorithm model, establish correlations between real-time wellhead temperature, tubing pressure, casing pressure, daily gas production, daily water production, and gas well conditions such as liquid loading and hydrate freezing (Fig 2). Through parameter iteration, split the dataset of 120 wells into training, testing, and validation sets, labeling each well’s operating conditions individually.

Step 1: To obtain valid production data (e.g., temperature, pressure, gas volume) and improve algorithm efficiency, perform data cleaning in advance. Apply iterative judgment methods to efficiently filter abnormal and redundant data. Combine preprocessing techniques such as normalization and standardization (see Formula 2-1) to eliminate dimensional differences and extract valid data for model input [4,5].


$$\hat{x}=(x-x_{\min})/(x_{\max}-x_{\min})$$ (2-1)

where X is raw data, X_min_ is the minimum value in the sequence and X_max_ is the maximum value in the sequence.

Step 2: Develop an identification method for low-efficiency wells and attribute parameter calculation models for wellbore and wellhead conditions.

Wellbore Issues (Liquid Loading): Key parameter: critical liquid-carrying flow rate. Based on the high salinity of formation water in the Sichuan-Chongqing exploration area, establish a liquid-loading identification model: If the unloading flow rate exceeds the actual daily gas production, liquid accumulation is confirmed. Unloading Flow Rate Calculation Model:

The unloading flow calculation model is [6]:


$$q_{cr}=2.5\times 10^{4}\times \frac{Apu_{cr}}{ZT}$$ (2-2)

$$u_{cr}=3.1\times {\left[\frac{\sigma g\left(\rho_{l}-\rho_{g}\right)}{\rho_{g}^{2}}\right]}^{0.25}$$ (2-3)

where, $q_{cr}$ is the discharge flow $\text{1}{\text{0}}^{4}{\text{m}}^{\text{3}}\text{/d}$; $u_{cr}$ is the unloading velocity m/s;A is the cross-sectional area of the oil pipe ${\text{m}}^{\text{2}}$;p is the pressure at a certain point in the tubing MPa;T is the temperature of the same position of the tubing K;Z is the gas deviation coefficient; $\rho_{g}$ is the gas phase density $\text{kg/}{\text{m}}^{\text{3}}$; $\rho_{l}$ is Liquid phase density $\text{kg/}{\text{m}}^{\text{3}}$.

Clarify Wellhead Issues: Hydrate Blockage, Key Parameters: Temperature and Pressure. Based on the characteristics of extremely cold weather in the Songliao old oilfield area, a hydrate blockage condition discrimination model has been established. The formation of hydrate blockage at the wellhead (typically at the surface pipeline location of gas wells) is closely related to low temperatures and high-pressure conditions. The critical pressure calculation formula is as follows:


logp=−1.0056+0.0541×(B+Ta−273),T>273 (2-4)

$$\log p=-1.0055+0.017\times \left(B_{1}-Ta+273\right),T\leq 273$$ (2-5)

where, *T*_*a*_ is the test point oil pipe temperature K;p is the critical pressure of hydrate formation corresponding to the temperature *T*_*a*_ at the test point MPa; B And $B_{1}$ is the parameter, $\gamma_{g}$ is determined by the value of the relative density of natural gas.

When T > 273 K, the temperature is relatively high, and the likelihood of hydrate formation is lower. When T ≤ 273 K, the temperature is relatively low, and the likelihood of hydrate formation is higher. In the formula, log p represents the logarithm of pressure, indicating a linear relationship between pressure p, temperature T, and parameters B or B_1_. Under low-temperature conditions (T ≤ 273 K), changes in pressure may directly affect hydrate formation. Higher pressure promotes hydrate formation, while lower pressure may inhibit it. Using the formula, the critical points for hydrate formation under different temperature and pressure conditions can be calculated, enabling the implementation of preventive measures to avoid blockage.

By calculating critical parameters (e.g., liquid-carrying flow rate, temperature, pressure) in the wellbore and wellhead, an innovative coupled wellbore-pipe flow and wellhead-throttling analysis model is developed. This model accurately describes pressure, temperature, and velocity fields across the entire flow domain, providing a theoretical foundation for root-cause analysis of inefficiencies.

Wellbore-Pipe Flow Model: Determines pressure gradient, temperature drop, and flow velocity.


$$\Delta {\text{p}}_{\text{w}}\;\left(i\right)=\Delta {\text{p}}_{\text{wj}}+\Delta {\text{p}}_{\text{accj}}+\Delta {\text{p}}_{\text{qz}}$$ (2-6)

where:

$\Delta p_{wj}$: Pressure drop due to friction and throttling.

$\Delta p_{\text{accj}}$: Pressure drop due to liquid acceleration and gravity.

$\Delta p_{\text{accj}}$: Pressure drop from gas-liquid two-phase flow and resistance.

Wellhead-Throttling Model: Calculates pressure and temperature drops across the choke.


$$q_{sc}=\frac{4.066\times 10^{3}p_{1}d^{2}}{\sqrt{r_{g}T_{1}Z_{1}}}\sqrt{\left(\frac{K}{K-1}\right)\left[{\left(\frac{p2}{p1}\right)}^{\frac{2}{K}}-{\left(\frac{p2}{p1}\right)}^{\frac{K+1}{K}}\right]}$$ (2-7)

where:

p1: Upstream pressure (MPa).

p2: Downstream pressure (MPa).

d: Choke diameter (m).

r_g_: Fluid density (kg/m^3^).

T: Fluid temperature (°C).

Z: Compressibility factor

K: Flow coefficient

**Fig 3:**
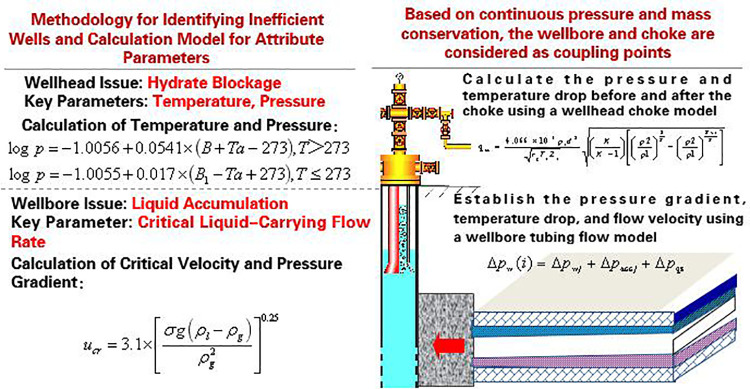
Schematic diagram of coupled flow and analysis between wellbore tubing flow and wellhead choke.

**Fig 4:**
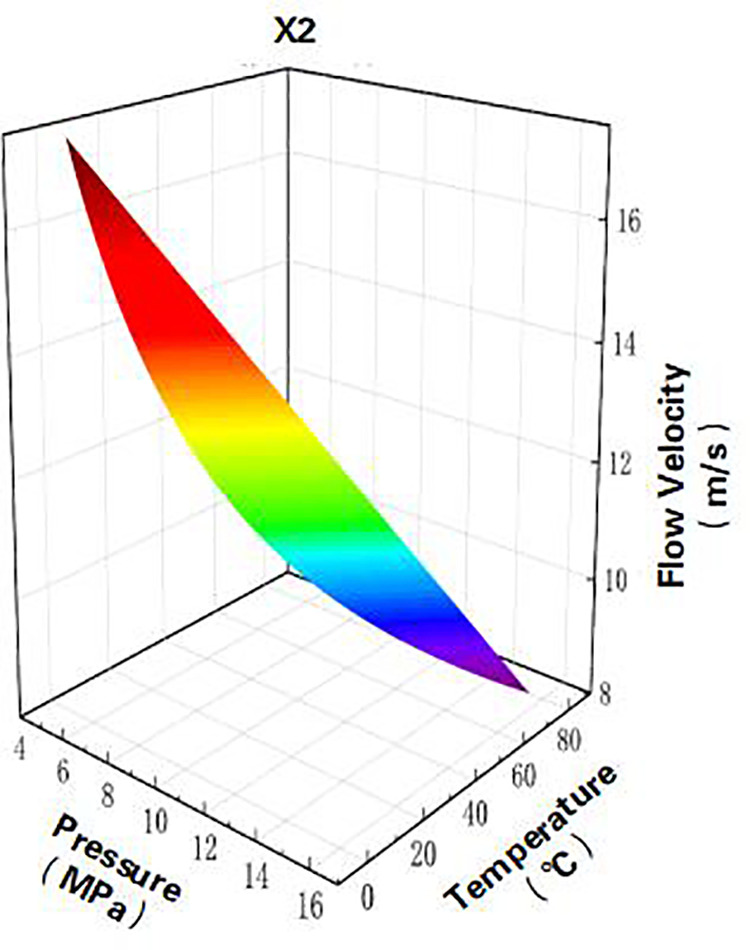
Pressure, temperature, and flow velocity fields after coupling.

Finally, coupling the wellbore and wellhead models generates a 3D pressure-temperature-velocity field, as illustrated in the Figs 3 and 4 below.

Step 3: Establishment of the GBDT (Gradient Boosting Decision Tree) Model. By integrating the liquid loading and hydrate freezing/blockage condition identification models, labels are assigned to the target values of “to-be-diagnosed” data in the operational diagnosis algorithm [7,8]. Empirical methods are then employed to identify patterns in operational condition variations across extensive training datasets. The training set completes model library construction, while the test set is used for model calibration and the validation set for model evaluation. The GBDT model development process is as follows:

(1) Build the initial model function$f_{0}\left(x\right)$:

$$f_{0}(x)=\mathrm{\backslash arg}\underset{c}{\min}\;\sum_{i=1}^{n}L(y_{i},c)$$ (2-8)

(2) Calculated negative gradient:

$$r_{ij}=-{\left[\frac{\partial L(y,f)}{\partial f^{\left(i\right)}}\right]}_{f=f_{j-1}}$$ (2-9)

(3) As $r={\left[r_{1j},r_{2j},\cdots ,r_{nj}\right]}^{T}$ a new target, a new model $h_{j}\left(x\right)$ is constructed on the training:
(4) Solving step size $s_{j}$:

$$s_{j}=\mathrm{\backslash arg}\underset{s>0}{\min}\;\sum_{i=1}^{n}L(y_{i},f_{j-1}(x_{i})+sh_{j}(x_{i}))$$ (2-10)

(5) Update function f to:

$$f_{j}(x)=f_{j-1}(x)+s_{j}h_{j}(x)$$ (2-11)

(6) Repeat step (2)–(5);
(7) Get the final model as follows:

$$f(x)=f_{m}(x)$$ (2-12)

Step 4: GBDT Model Application for Low-Efficiency Zone Identification. The GBDT model processes extensive test datasets, integrating computational results with benchmark characterization metrics. This enables precise identification of low-efficiency zones and localization of operational root causes (“where issues exist”) (Table 1). By correlating model outputs with field performance indicators, the system diagnoses inefficiency origins, providing actionable insights for targeted interventions.

**Table 1:** Classification criteria for low-efficiency well subcategories.

Low-Efficiency Causes	Category	Characterization Metrics
**Wellhead**	Hydrate Freezing/Blockage	Tubing pressure rise rate: > 0.5 MPa/h Temperature: Deviates from normal trend
	Production Shutdown Due to Blockage	Flowline pressure drops to system pressure
**Wellbore**	No Liquid Loading	Pressure gradient: < 0.25 MPa/100m Critical velocity ratio ≤1
	Mild Liquid Loading	Pressure gradient: 0.25–0.4 MPa/100m Critical velocity ratio: 1 < ratio ≤1.25
	Moderate Liquid Loading	Pressure gradient: 0.4–0.6 MPa/100m Critical velocity ratio: 1.25 < ratio ≤2.5
	Water Invasion	Pressure gradient: > 0.6 MPa/100m Critical velocity ratio: > 2.5

**Fig 5:**
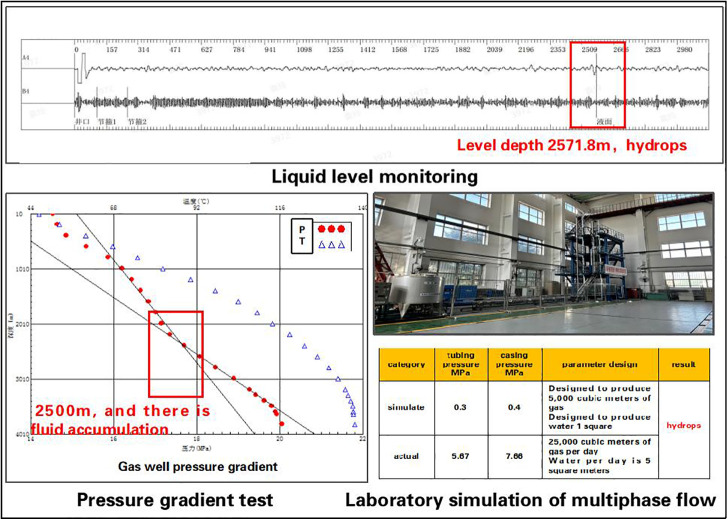
Accuracy verification of big data diagnosis and early warning algorithm.

A validation study was conducted on 120 wells using the operational condition diagnosis and early-warning algorithms, achieving an accuracy rate of 92%. For instance, the big-data-driven diagnosis algorithm identified liquid loading in Well A1 located in the Songliao Basin (Fig 5). Subsequent validation through multiphase flow simulation experiments, field liquid level monitoring, and pressure gradient testing confirmed a 100% diagnostic accuracy, fully aligning with actual production conditions. This demonstrates the algorithm’s robust capability to precisely reflect real-world operational scenarios.

### 2.2. Research on optimization technology for adjusting parameters of gas wells under multiple operating conditions

**Fig 6:**
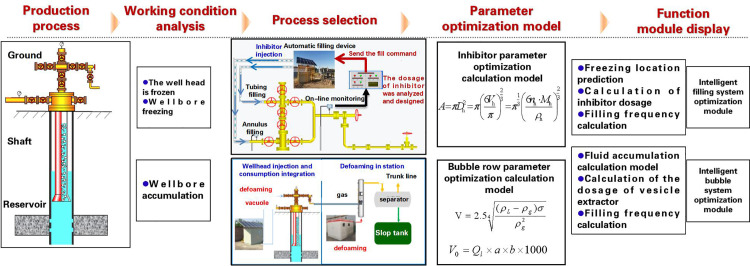
Measure parameter optimization and remote measurement and control technology route.

Real-time production data are comprehensively acquired through front-end digital devices to accurately evaluate gas well production status and conduct multidimensional diagnosis of downhole-surface operational conditions [9–12]. Combined with a condition early-warning mechanism, this supports process selection and parameter optimization for auxiliary measures, ultimately providing decision-making support for low-efficiency well management. A real-time parameter adjustment optimization methodology for de-freezing/unblocking and foam drainage measures is established, enabling closed-loop control of automated chemical injection systems through remote monitoring and control technologies (Fig 6).

#### 2.2.1. Establishment of a parameter optimization model for foam drainage gas recovery in liquid-loaded wells.

Liquid Loading Well Optimization: Foam Drainage Parameter Injection Model. For liquid-loaded wells, a foam drainage parameter injection optimization model is developed to effectively guide downhole drainage operations. This includes:

(1) Foaming Agent Concentration Calculation Model

Establishes correlations among chemical dosage concentration, surface tension, and minimum liquid-carrying velocity to determine cost-effective dosage (Q). This model minimizes operational impacts on surface facilities while reducing chemical costs [13–17].


$$Q=2.5\sqrt[{4}]{\frac{\left(\rho_{L}-\rho_{g}\right)\sigma }{{\rho^{2}}_{g}}}$$ (2-13)

where:

Q——Concentration of vesicle extractor, ‰;

*ρ*_*L*_——Density of effervescent agent, kg/m^3^;

*ρ*_*g*_——Density of water, kg/m^3^;

σ——Surface tension after dosing, N/m.

High-Salinity Well Solutions: A novel nano-foaming agent system (Table 2) is developed for high-salinity, low-efficiency liquid-loaded wells in Sichuan-Chongqing regions, addressing diverse formation environments.

**Table 2:** Concentration application criteria for high-salinity-resistant foam drainage agent systems.

Model	Formation Water Parameters	Dosage Concentration Q (‰)
1#	Salinity: < 20 × 10⁴mg/L; Condensate oil: < 10%	3 ~ 5
2#	Salinity:20–25 × 10⁴mg/L; Condensate oil: < 20%	5 ~ 7
3#	Salinity:25–35 × 10⁴mg/L; Condensate oil: < 25%	5 ~ 9

(2) Calculation model of total water yield

The calculation model of total water production is built to determine the total water production a in the wellbore. The total water production consists of two parts, one is the amount of fluid accumulated in the wellbore and the other is the amount of water produced before the measure, and the sum of the two is the total water production of the well.


$$a=V_{1}+V_{2}$$ (2-14)

where:

a——Gross water yield, m^3^;

V_1_——The amount of fluid accumulated in the wellbore, m^3^;

V_2_——Pre-measure water yield, m^3^.

(3) The optimization model of bubble row parameter filling was constructed

The filling optimization model of bubble discharge parameters is as follows: total daily injection amount = daily water volume×concentration of bubble discharge agent used×filling frequency [18–21]. The total daily injection amount can be obtained by using concentration of bubble discharge agent Q, daily water volume a and filling frequency b (manually entered remotely).


$$V_{0}=Q_{l}\times a\times b\times 1000$$ (2-15)

#### 2.2.2. Establishment of optimization model of plugging removal measures for freezing blocked wells.

Aiming at hydrate-frozen plugging, the optimization model of plugging removal parameters is established to effectively guide the implementation of plugging removal technology at wellhead and wellbore. The transient calculation model of wellbore pipe flow was established to accurately describe the working conditions of hydrate formation, and the formation and decomposition model of natural gas hydrate was introduced to realize the quantitative prediction of hydrate formation risk [22–25].

(1) Wellbore gas-liquid mixing momentum model (ρdensity(kg/m^3^), v phase velocity (m/s), E potential energy (J), Ddiameter (m)):

$$\begin{array}{cc} & \frac{\partial }{\partial t}(\rho_{l}v_{l}E_{l}+\rho_{g}v_{g}E_{g}+\rho_{s}v_{s}E_{s}\text{)}\;\text{+}\\ & \frac{\partial }{\partial \text{z}}(p_{a}+\rho_{l}v_{l}^{2}E_{l}+\rho_{g}v_{g}^{2}E_{g}+\rho_{s}v_{s}^{2}E_{s})+\\ & (\rho_{l}E_{l}+\rho_{g}E_{g}+\rho_{s}E_{s})g\sin\theta +\frac{\lambda \rho_{a}v_{a}^{2}}{2(D_{ci}-D_{po})}=0 \end{array}$$ (2-16)

(2) Theoretical model of wellbore pressure field (P pressure (MPa)):

$$\frac{dP_{m}}{dz}=\frac{dP_{g}}{dz}+\frac{dP_{f}}{dz}+\frac{dP_{a}}{dz}$$ (2-17)

(3) Theoretical model of wellbore temperature field (T temperature (°C), c Specific heat capacity at constant pressure (J/(kg·K)), q Heat exchange capacity (J/s)):

$$\frac{dT_{m}}{dz}=\frac{-4}{\rho_{\text{m}}v_{m}c_{m}\pi D_{pi}^{2}}\left(q_{f}+q_{h}+q_{e}\right)$$ (2-18)

(4) Model of formation and decomposition of natural gas hydrate:

$$\frac{dn_{f}}{dt_{f}}=K_{f}A_{f}\left[f_{m}\left(T_{m},P_{m}\right)-f_{eq}\left(T_{m},P_{eq}\right)\right]$$ (2-19)

$$-\frac{dn_{d}}{dt_{d}}=K_{d}A_{d}\left[f_{eq}\left(T_{m},P_{eq}\right)-f_{m}\left(T_{m},P_{m}\right)\right]$$ (2-20)

(5) Hydrate formation/decomposition reaction area:

$$A=\pi D_{h}^{2}=\pi {\left(\frac{6V_{h}}{\pi }\right)}^{\frac{2}{3}}=\pi^{\frac{1}{3}}{\left(\frac{6n_{h}\cdot M_{h}}{\rho_{h}}\right)}^{\frac{2}{3}}$$ (2-21)

**Fig 7:**
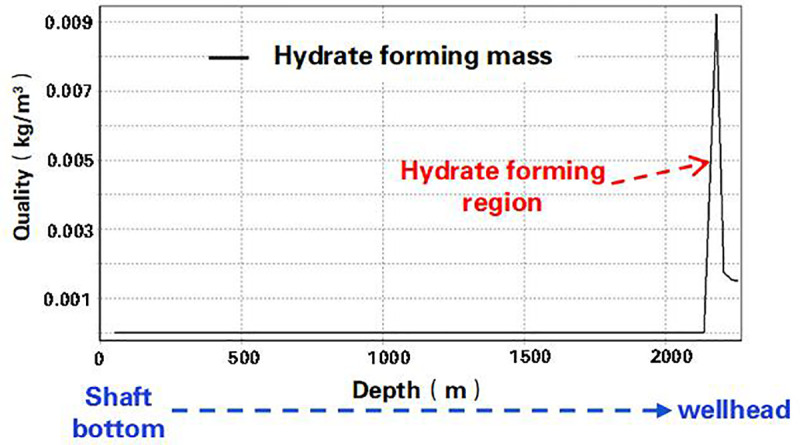
Prediction curve of hydrate formation position.

**Fig 8:**
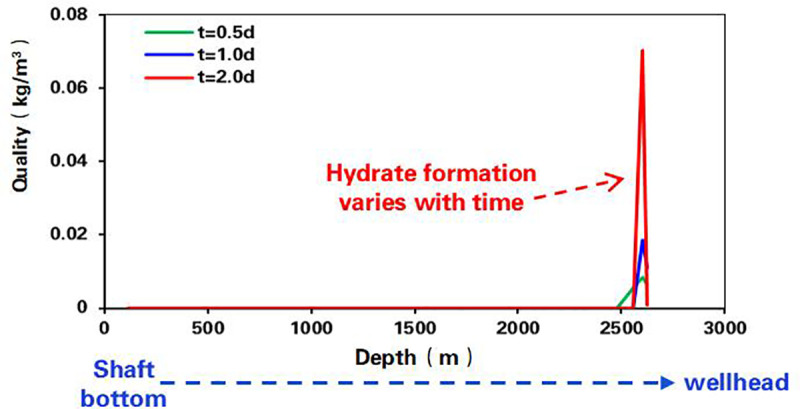
Hydrate formation time prediction curve.

Considering gas well working conditions and natural gas components, based on the hydrate thermodynamic and dynamic generation model and the transient calculation method of wellbore multiphase flow and temperature field, the time term was introduced to establish a dynamic prediction model of hydrate generation to predict the location, timing and quantity of hydrate generation, so as to determine the methanol injection amount (Figs 7 and 8). By determining the injection frequency, the optimal injection working system was established [26–28].

**Fig 9:**
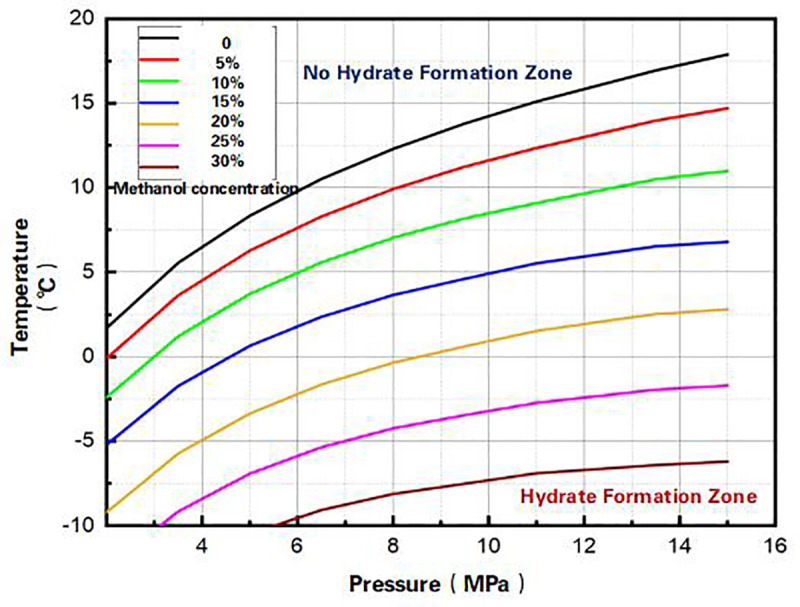
Wellhead methanol injection critical concentration discrimination chart.

**Fig 10:**
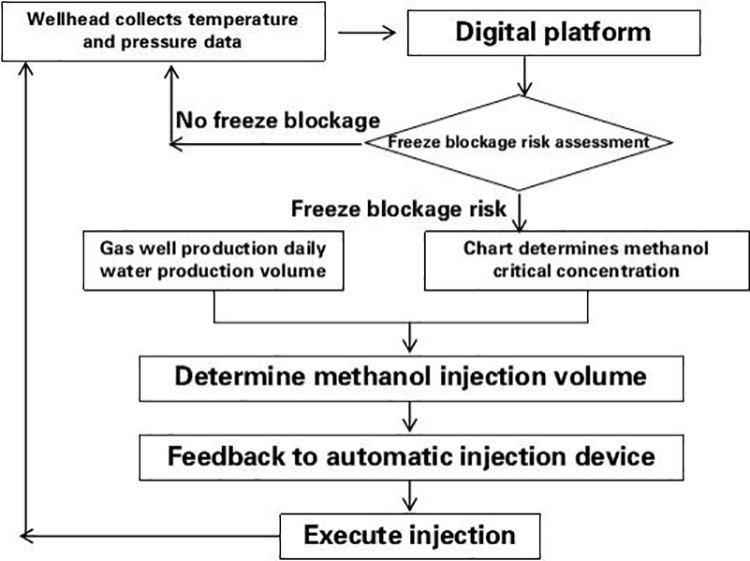
Methanol injection system optimization closed-loop process.

Hydrate Freezing Prevention and Methanol Injection Optimization. Hydrate prediction models prevent issues like “freezing blockage” and “post-blockage remediation.” Laboratory simulations of methanol injection establish critical concentration charts (Fig 9). Real-time monitoring and freezing risk analysis determine optimal methanol injection timing and volume [29,30], enabling precise automated dosing to avoid well shutdowns during extreme cold (operational workflow in Fig 10).

**Fig 11:**
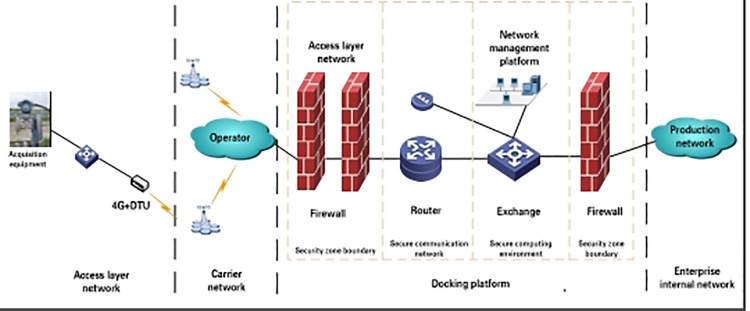
Establish a data transmission channel.

Integrated Analysis and Remote Monitoring Platform. A five-layer platform architecture (user, application, data, network transmission, and control layers) with seven functional modules enables data upload and command execution (Fig 11). For widely dispersed wells in Field A with harsh environments and high reliability demands, a 4G+VPDN network ensures real-time communication (packet loss <5%, RSRQ = -11dB) [31–33].

## 3. Field test and application effect

**Fig 12:**
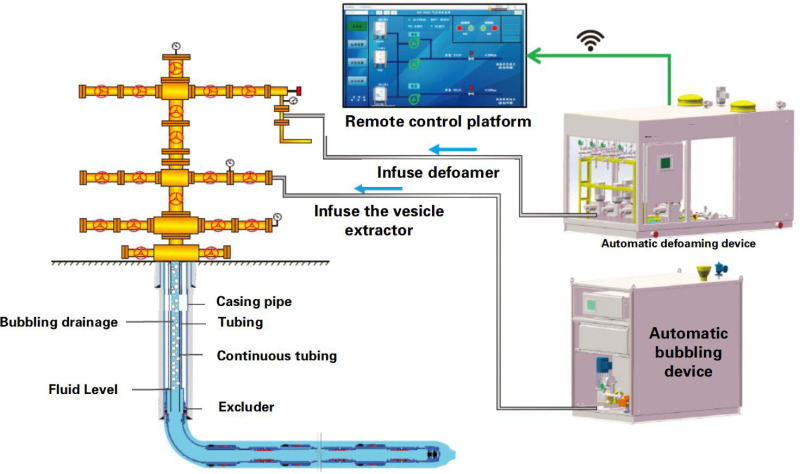
Digital transformation diagram of well X1.

**Fig 13:**
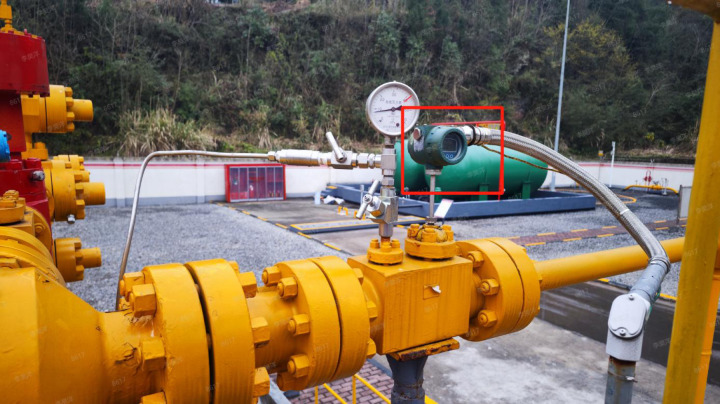
X1Well site digital instrument.

**Fig 14:**
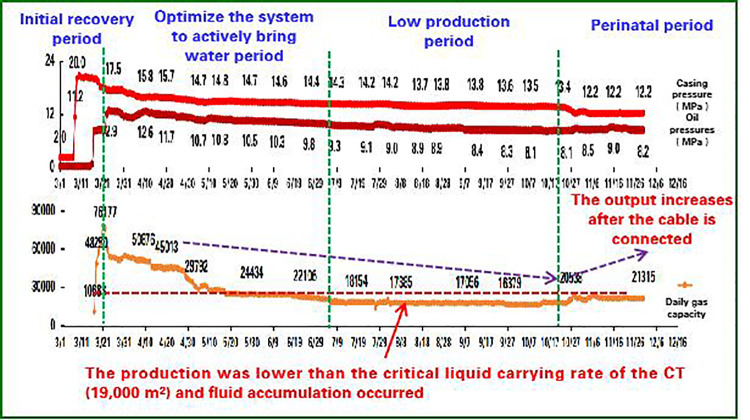
Production curve of well X1.

Well X1: Post-waterflooding restart, digital monitoring enabled real-time pressure analysis and foam drainage optimization (Figs 12–14). Automated dosing adjusted production parameters, increasing daily gas output from 17,000–22,000m^3^, sustaining 400 days with 9 million m^3^ incremental production.

**Fig 15:**
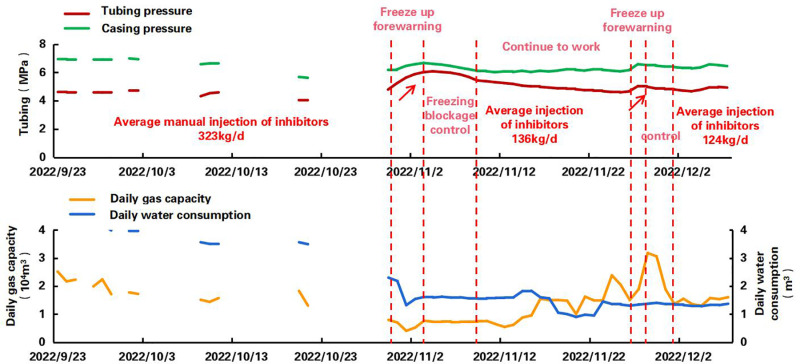
Production curve of well X2.

Well X2: Digitization replaced manual methanol injection for hydrate management, optimizing dosage and injection schedules (Fig 15). Well uptime improved from 23.4% to 100% with reduced chemical consumption [34,35].

In 2023, Field A achieved 100% success in digital management trials across 24 low-efficiency wells, yielding 22 million m^3^ incremental gas and 21 million economic benefits. Planstar get 100 wellsover five years, establishing digital demonstration zones inSongliao and Sichuan for > 21 million economic benefits. Planstarget 100 wellsover fivey ears, establishing digital demonstration zones in Song liao and Sichuan for > 80 million returns.

## 4. Discussion

In this study, we addressed the issues of hydrate blockage caused by low temperatures in extremely cold weather and poor foam drainage performance due to high formation water salinity by proposing digital-based control technologies. The application effects were systematically analyzed and summarized as follows:

To tackle the problem of hydrate blockage caused by low temperatures in extremely cold weather, we established a digital-based “blockage prediction + precise chemical injection” control technology. This technology can accurately locate the formation and quantity of hydrates. Combined with a wellhead continuous injection device, it optimizes the injection volume and frequency of hydrate inhibitors, transforming the hydrate removal measures from a traditional “passive treatment” mode to a “sustained and effective” proactive management mode. This significantly improves the efficiency and effectiveness of hydrate prevention and control.

To address the issue of poor foam drainage performance caused by high formation water salinity, we developed a digital-based “foam drainage injection parameter optimization” control technology. This technology defines key parameters such as the injection concentration, ratio, dosage, and frequency of high-salinity-resistant foam drainage agents. Combined with a wellhead integrated “injection + defoaming” device, it enables continuous and precise injection of foam drainage and defoaming agents. This technology shifts foam drainage measures from “passive response” to “active optimization,” significantly enhancing the adaptability and efficiency of foam drainage processes.

Despite the significant achievements of the above technologies, current research still has certain limitations. At present, digital control technologies have only been successfully applied to foam drainage and hydrate removal processes, while other process measures have not yet been deeply integrated with digital technologies. Additionally, the low quality of real-time production data remains a major bottleneck affecting the accuracy of working condition diagnosis and parameter adjustment, necessitating further optimization of data collection and processing technologies.

Based on the three-step strategic deployment of “digital gas field, intelligent gas field, and smart gas field,” future research needs to further deepen the study of digital control technologies for low-efficiency gas wells, expand the application areas of these technologies, and fill technical gaps to meet the measures required throughout the entire lifecycle of gas fields. Through continuous technological innovation and optimization, labor costs can be reduced, work efficiency improved, and gas field management steadily advanced toward smartization.

In summary, this study provides effective digital solutions for gas well management under extremely cold weather and high-salinity formation conditions, while also pointing out future research directions and priorities, laying a technical foundation for the construction of smart gas fields.
