# Correction: Research and application of digital measure management and control technology for characteristic low-efficiency gas wells in Gas Field A

**DOI:** 10.1371/journal.pone.0327060

**Published:** 2025-06-25

**Authors:** Hao-yang Li, Wen-hai Ma, Jun-liang Li, Pin-gang Ma, De-song Yao, Ming-xi Feng, Shao-xing Gu

The author Cheng-gang Jiang should not have been included in the author byline.

The correct citation is: Li H-y, Ma W-h, Li J-l, Ma P-g, Yao D-s, Feng M-x, et al. (2025) Research and application of digital measure management and control technology for characteristic low-efficiency gas wells in Gas Field A. PLoS One 20 (5): e0323644. https://doi.org/10.1371/journal.pone.0323644.
